# Poster Session I – Poster of Distinction A94 AUTOMATING POST-ENDOSCOPY FOLLOW-UP VIA SHORT MESSAGE SERVICE (SMS): EFFECTIVENESS AND FEASIBILITY

**DOI:** 10.1093/jcag/gwaf042.094

**Published:** 2026-02-13

**Authors:** M Ten-Pow, L Calman T’ien, C Galorport, J J Telford, R A Enns

**Affiliations:** St. Paul’s Hospital, Division of Gastroenterology, Vancouver, BC, Canada; The University of British Columbia Faculty of Medicine, Vancouver, BC, Canada; St. Paul’s Hospital, Division of Gastroenterology, Vancouver, BC, Canada; St. Paul’s Hospital, Division of Gastroenterology, University of British Columbia, Vancouver, BC, Canada; St. Paul’s Hospital, Division of Gastroenterology, University of British Columbia, Vancouver, BC, Canada

## Abstract

**Background:**

Telehealth advancements through the short message service (SMS) have been increasingly integrated into clinical care, validated in appointment management, disease surveillance, and preventive health. With the ubiquity of mobile phones, SMS offers a scalable channel for patient communication and can inform quality improvement. However, its application to post-endoscopy follow-up and adverse-event reporting remains understudied. Given the imperative to detect post-endoscopy complications, research is warranted to evaluate SMS initiatives as a low-burden and cost-effective modality for complication tracking.

**Aims:**

To evaluate the design effectiveness and feasibility of an automated follow-up program via SMS communication.

**Methods:**

An iterative prospective study evaluated a PAtient-Guided Complication Tracking System (PACTS) administered at St. Paul’s Hospital, Vancouver (Sept–Oct 2025). Outpatients undergoing colonoscopy, gastroscopy, or flexible sigmoidoscopy with mobile phone access were eligible to participate. PACTS delivered a one-time SMS prompt, 7 days post-endoscopy; patients replied with numeric responses to complete follow-up. Those with complications were digitally routed to a survey for event classification. Ambient clinic signage and PACTS discharge leaflets were disseminated to support enrollment and minimize staff involvement. Brief clerical introductions, consistent sender IDs, and hyperlink-free initial SMS communications were implemented to support sender verification and message legitimacy. No reminder SMS was issued during the pilot. Program effectiveness was determined via SMS response rates, delivery success, and staff time per 100 patients. Two-sided 95% CIs were computed via the Wilson score (binomial) method.

**Results:**

Among 567 eligible outpatients, 524 (92%) SMS were successfully delivered; 383 replies yielded an overall response rate of 73% (95% CI: 0.69–0.77) and a daily median response rate of 71% (IQR 65–74%). 5 respondents (1%) reported post-endoscopy complications requiring follow-up or emergency room evaluation. Accounting for clerical project introductions and PACTS discharge leaflet dissemination, staff effort was approximately 12 minutes per 100 patients, indicating minimal operational burden.

**Conclusions:**

PACTS, an SMS-based post-endoscopy follow-up initiative, achieved appreciable engagement requiring minimal staff time, supporting feasibility and scalability in routine practice. Ambient engagement materials at admission and discharge likely enhanced responsiveness. Future research should identify behavioural and contextual barriers in populations wary of receiving unfamiliar SMS and evaluate reminder messages to non-respondents to optimize reach and response.

**Funding Agencies:**

St. Paul’s Foundation – Enhanced Patient Care Grant

